# Compound kushen injection for multiple myeloma: A protocol of systematic review and meta-analysis

**DOI:** 10.1097/MD.0000000000018257

**Published:** 2019-12-16

**Authors:** Xinwei Yang, Xiyun Zhao, Xiaoxia Wang

**Affiliations:** a Department of Hematology, Gansu Provincial Hospital of Traditional Chinese Medicine; b Affiliated Hospital of Gansu University of Traditional Chinese Medicine; c Gansu Gem Flower Hospital, Lanzhou, China.

**Keywords:** compound kushen injection, efficacy, meta-analysis, multiple myeloma, safety

## Abstract

**Background:**

Compound kushen injection is increasingly used to treat various cancers. However, its role in the management of multiple myeloma (MM) is still controversial and requires further clarification. The aim of this study is to evaluate efficacy and safety of compound kushen injection for MM through systematic review and meta-analysis.

**Methods:**

We are going to search the 6 electronic databases: PubMed, Embase, Cochrane Library, China National Knowledge Infrastructure, Wangfang and Chinese Biomedical Literature Database. General characteristics and Specific trial characteristics will be collected from the included studies. The outcomes included overall response rate, complete response rate, 3-year progression—free survival rate, 3-year overall survival rate, and different types of treatment-related adverse events. We calculated the risk ratios as well as their 95% confidence intervals of these outcomes and pooled the results through RevMan 5.2 software.

**Discussion:**

The results of the study will be submitted to a peer-reviewed journal for publication.

**Ethics and dissemination:**

This study is not a clinical study, ethical approval is not required.

## 1 Introduction

Multiple myeloma (MM), a complex and serious disease results from a proliferation of plasma cells often accompanied with osteolytic lesions, renal failure, anemia, and hypercalcemia, is the second most frequent hematologic malignancy around the world, accounted for approximately 10% of all hematologic malignancies.^[1]^ In Western countries, the incidence is about 5.6 cases per 100,000 people, with a median age at diagnosis being 70 years and a third of patients over age 75.^[2]^ Although MM currently remains an incurable malignancy, the treatments for MM has been greatly increased in the last decade, with novel proteasome inhibitors (PIs), immunomodulatory drugs (IMiDs). And monoclonal antibodies now are being incorporated in first-line treatment regimens, which have considerably improved progression-free survival (PFS) and overall survival (OS) of MM.^[3–5]^ More and more patients with MM benefit from these new treatments. However, chemotherapy, especially high-dose chemotherapy, both PIs and IMiDs have different adverse events (AEs) for patients with MM.^[6–8]^ These AEs can usually lower the quality of life and increase burden of patients. According to available evidence, for example, the combination of traditional Chinese medicine and chemotherapy has a promising clinical effect on patients with MM prolonging the patient's life and improving the quality of life.^[9,10]^

Compound kushen injection (CKJ) as a Chinese patent medicine was approved for treating cancer, inflammatory disease, and pain, etc. by the State Food and Drug Administration of China. CKJ consists of the following 2 Chinese herbs: sophora flavescens and polygonum sibiricum. The combination of the 2 Chinese herbs promotes eliminating stagnation stop pain, strengthens and consolidates body resistance, clears heat, and removes toxicity.^[11]^ A alkaloids, fatty acids, and phenolic acids, are the main active compounds in CKJ, and CKJ has been widely used in the adjuvant treatment of severe diseases such as non-small cell lung cancer, primary liver cancer, digestive tract cancer, MM and malignant pleural effusion.^[12–14]^ Given that both the number and quality of clinical trials using CKJ to treat MM have improved considerably in recent years, we sought to assess its efficacy and safety through a meta-analysis.

## 2 Methods

The content of this protocol follows the preferred reporting items for systematic review and meta-analysis protocols (PRISMA-P) recommendations.^[15]^ This review has been registered on the International Prospective Register of Systematic Reviews,^[16]^ with the identification number CRD42019133414. If protocol amendments occur, the dates, changes, and rationales will be tracked in the International Prospective Register of Systematic Reviews.

### 2.1 Criteria for inclusion and exclusion in the present review

#### 2.1.1 Types of participants

Patients who aged 18 years or older were diagnosed MM by pathology or cytology, regardless of whether the patient has received treatment before. There are no restrictions on gender, race, etc. Patients accompanied by other malignant tumor or non-primary MM will not include.

#### 2.1.2 Types of interventions

CKJ maintenance therapy vs placebo, or CKJ combined others treatments (such chemotherapy, radiotherapy, etc.) vs others treatments (others treatments of these comparable group must be the same).

#### 2.1.3 Types of outcome

Outcomes of this meta-analysis will include overall response (OR), complete response (CR), 3-year OS, 3-year PFS and treatment-related AEs. OS is defined as the time from the date of randomization to death from any cause. PFS is defined as the time from the date of randomization until disease progression or death. As for AEs, we will analyze the Grade 3 or 4 hematological and nonhematological toxicity.^[17]^

#### 2.1.4 Types of studies

Randomized controlled trials (RCTs) of CKJ for MM will be included to pool and review in this study. Non-RCTs, observational studies, qualitative studies, and laboratory studies will not be considered.

### 2.2 Search strategy

Search strategy will be performed on 6 electronic databases: PubMed, Embase, Cochrane Library, China National Knowledge Infrastructure, Wangfang and Chinese Biomedical Literature Database. The MeSH search and text word will be used with the terms related to MM and compound kushen. We plan to search these databases from inception to April 2019, and the publications language will be restricted as English and Chinese. The search strategy is shown in Table 1 that taking PubMed as an example, and will be modified for other databases use if necessary. Other potentially eligible studies will also be manually searched the reference lists from the trials identified.

**Table 1:**
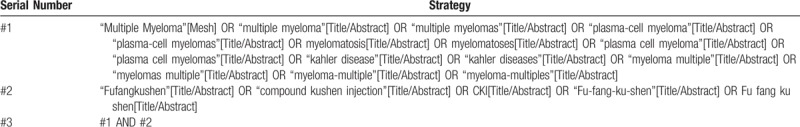
Searching strategy in PubMed.

### 2.3 Study selection

**Figure 1:**
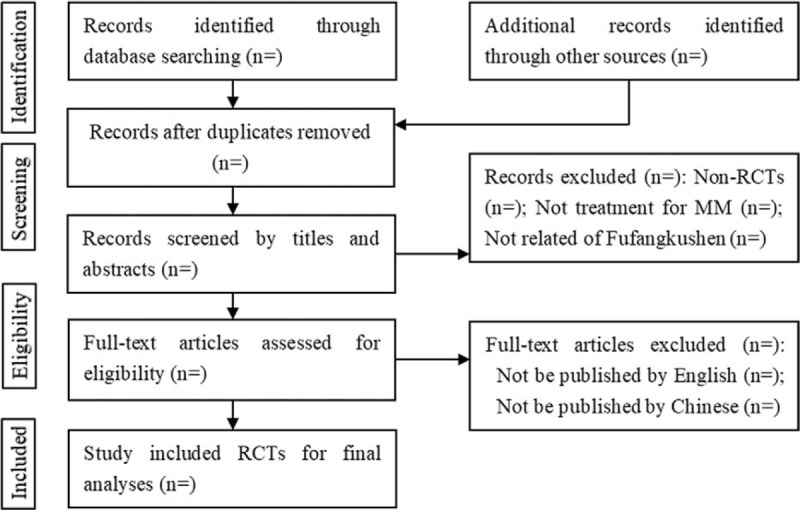
Study selection for meta-analysis. MM= multiple myeloma; RCTs= randomized controlled trials.

All initial records from six electronic databases will be imported into the EndNoteX8 software (Thomson Reuters [Scientific] LLC, Philadelphia, PA). First, the titles and abstracts of all records will be reviewed independently by 2 reviewers. Then, full text of all potentially relevant articles will be retrieved to make the final decision. Any conflict will be resolved by discussion. A flow diagram will be used to describe the process of RCTs selection (Fig. 1).

### 2.4 Data collection

We plan to extract the following items from the included RCTs:

(i) General characteristics: author, year of publication, country where the study was performed, funding, study duration, contact details of the authors and identifiers;
(ii) Specific trial characteristics: sequence generation, allocation sequence concealment, blinding, incomplete outcome data, and selective outcome reporting, participants (total number, setting, age, sex, country, diagnostic criteria for MM, etc.), details of all experimental and control interventions (manufacturer of the drugs, dosages, medication route, duration of treatment, etc.), outcomes (OR, CR, OS, PFS, AEs);
(iii) We are also going to collect key conclusions, comments, and any explanations provided for unexpected findings by the study authors if applicable.

The steps of data collection: First, data extraction form will be designed based on previously identified items by our team; Second, 1 to 5 included studies will be pre-extracted, the forms shall be continually modified until the final data extraction form complete; Third, we shall contact the lead authors of included studies if there are issues to be clarified. Two reviewers will independently extract data from each included study. Different opinions will be resolved through discussion or consult the third part.

### 2.5 Quality assessment, risk of bias, and evidence profile

All data will be checked for internal consistency, and disagreements also be resolved by discussion. The quality of each clinical trial will be evaluated using the modified Jadad quality scores, including the presence of randomization, allocation concealment, blinding, and withdrawal/dropout.^[18]^ A general quality score was assigned to each study as follows: low quality studies (1–3), and high quality studies (4–5).

The risk of publication bias will be assessed by applying the funnel plot and Egger test. Evidence of asymmetry from Egger test will be considered to be significant at *P* < .1, and the graphical representation of 90% confidence bands is presented.^[19]^

The quality of the evidence obtained for the primary endpoints will be assessed in agreement with the Grading of Recommendations Assessment, Development, and Evaluation (GRADE) system.^[20]^

### 2.6 Data synthesis

Statistical analyses will be performed using Review Manager 5.3 statistical software (Cochrane Collaboration, Denmark). The outcomes will be presented as the relative risk, mean difference or standardized mean difference and its 95% confidence interval (95% CI). The statistical significance will be assessed for *P* < .05, and moderate to high levels of heterogeneity will be considered for I^2^ > 50%.^[21]^ A fixed effects model will be used if no statistical heterogeneity across the studies; otherwise, the random effects model will be considered.

### 2.7 Subgroup analysis

If sufficient studies are available, we are going to conduct subgroup analyses to explore the difference ages, doses, tumor staging, etc.

## 3 Discussion

Tradition Chinese medicine injection has been widely used in cancer, which effectiveness has been confirmed. However, the efficacy and safety of CKJ for MM is unclear. Therefore, we believe the results of this meta-analysis will provide some references for clinicians and researcher.

## Acknowledgments

We thank Ming Liu, Lanzhou university basic medical college, for registered this review on the International Prospective Register of Systematic Reviews.

## Author contributions

**Conceptualization**: Xinwei Yang, Xiaoxia Wang

**Funding acquisition**: Xiyuan Zhao.

**Methodology:** Xinwei Yang, Xiyuan Zhao

**Methodology:** Xiyun Zhao.

**Project administration**: Xinwei Yang, Xiaoxia Wang

**Writing – original draft**: Xinwei Yang, Xiaoxia Wang

**Writing – review & editing**: Xinwei Yang, Xiaoxia Wang.
